# Natural Strategies to Preserve Alcohol-Free Beer: Phenolamide Dimers with Anti-Yeast Potential

**DOI:** 10.1021/acs.jafc.5c09118

**Published:** 2026-02-22

**Authors:** Annemiek van Zadelhoff, Denise Dozio, Francesca Annunziata, Aziza Caccia, Sabrina Dallavalle, Yingyu Zhou, Sarah van Dinteren, Jean-Paul Vincken, Andrea Pinto, Wouter J. C. de Bruijn

**Affiliations:** † Laboratory of Food Chemistry, 4508 Wageningen University, Bornse Weilanden 9, Wageningen 6708 WG, The Netherlands; ‡ Department of Food, Environmental and Nutritional Sciences (DeFENS), 9304 University of Milan, Milan 20133, Italy

**Keywords:** hydroxycinnamic acid amides, antimicrobial activity, antiyeast activity, oxidative coupling, (neo)lignanamides, diferulates

## Abstract

Trends in clean label food and the growing popularity of alcohol-free beers have led to a demand for natural compounds to prevent microbial spoilage. Oxidative coupling products of hydroxycinnamic acids and their amides, i.e. phenolamides, obtained via chemo-enzymatic synthesis and from barley rootlets, a beer brewing byproduct, were tested for their inhibitory activity against the beer spoilage yeast *Saccharomyces cerevisiae* subsp. *diastaticus.* Hydroxycinnamic acids, their dimers, and hydroxycinnamoylagmatines (HCAgms) did not inhibit the yeast. However, inhibitory activity was observed with HCAgm dimers. In particular, CouAgm-4-*O*-7′/3-8′-DCouAgm and FerAgm-4-*O*-7′/3-8′-DFerAgm were active at 46–255 μg/mL in beer. Notably, the newly synthesized agmatine amide of poacic acid, a stilbenoid dimer of ferulic acid, showed increased antiyeast activity compared to its monomeric precursors. These results demonstrate that amidation and oxidative coupling can increase the antiyeast activity of hydroxycinnamic acid derivatives, and that hydroxycinnamoylagmatine dimers possess potential as preservatives against wild yeast spoilage in beer.

## Graphical abstract

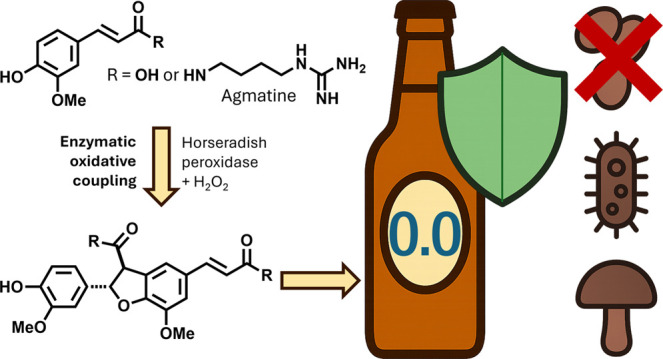

## 1 Introduction

The ongoing trend in clean label food, along with the growing popularity of alcohol-free and low-alcohol beers, pose new challenges for food preservation.^1^ No or limited alcohol can result in an increased sugar content, creating an ideal environment for proliferation of spoilage organisms.^2–4^ During malting, the first step in the beer production process, there is a high risk of fungal growth, especially of *Aspergillus, Penicillium,* and *Fusarium* strains.^5–8^ Throughout the brewing process and during subsequent storage, the main spoilage organisms are lactic acid bacteria (LAB), most commonly *Levilactobacillus brevis,* and wild yeasts (i.e., yeasts other than brewing yeast).^8–10^ Among them, the wild yeast *Saccharomyces cerevisiae* subsp. *diastaticus* is responsible for huge economic losses in the beer production chain, as it produces an increased amount of carbon dioxide resulting in gushing of beer and bottles bursting, as well as threatening the quality of alcohol-free and low-alcohol beer by triggering undesired alcohol formation.^11^ In order to avoid spoilage related to the proliferation of these organisms, preservatives could be added. However, to meet consumers’ demands for clean label food, these preservatives should be of natural origin, preferably plant-derived. Plants produce secondary metabolites as self-defense mechanism against biological or environmental threats, thus these metabolites can possess a wide array of biological activities, including antiyeast activity. Consequently, these secondary metabolites are interesting to explore as potential preservatives for food products, including beer, especially molecules derived from hops and barley, or more specifically barley malts: the two main ingredients of beer.^12,13^ Among hop secondary metabolites, hop bitter acids are well-known to inhibit the growth of Gram-positive bacteria, while some lactic acid bacteria have developed resistance against these compounds.^8,14^ On the other hand, the antimicrobial activity of various barley secondary metabolites has been much less extensively studied. Barley malt as used for brewing is the product of germination, a process resulting in alteration of the secondary metabolite profile and, thus, the presence of (potentially) antimicrobial compounds.^15^

Among barley secondary metabolites, the class of phenolamides and their dimers, called (neo)­lignanamides, are part of the barley defense response. Their accumulation, especially in shoots,^16^ is triggered by biotic or abiotic stress factors.^16–18^ For example, an upregulation of phenolamides and (neo)­lignanamides was observed in barley after infection with powdery mildew (*Blumeria graminis* f.sp. *hordei*) or upon a 5 °C increase in temperature (from 19 to 24 °C).^19^

Hydroxycinnamoylagmatine (HCAgms) are phenolamides obtained by amidation of a hydroxycinnamic acid with agmatine.^20,21^ Their biosynthesis in plants is catalyzed by the enzyme agmatine coumaroyltransferase (ACT), which is responsible for the conjugation of agmatine to *p*-coumaroyl-CoA or feruloyl-CoA.^22^ Coumaroylagmatine (CouAgm) is described to have a moderate antimold activity as inhibitor of spore formation against *Monilinia fruticola* and *Erypsiphe graminis* (400 μM and 72 mM respectively),^20,22^ while feruloylagmatine (FerAgm) seems to be relatively ineffective as an inhibitor of spore germination (Table 1).^21^ However, Dozio et al. recently reported that FerAgm inhibits *Pyricularia oryzae* appressoria formation, the fungus’ main infection mechanism, with 94% inhibition at 500 μM.^23^

**Table 1:** Overview of the Reported Antimold Activities of Hydroxycinnamoylagmatines and Their Oxidative Coupling Products from Barley

fungal strain	compound tested^a^	spore formation inhibiting concentration
*Monilinia fructicola*	CouAgm	400 μM (122.4 μg/mL)^34^
	Hordatine A(CouAgm-4-*O*-7′/3-8′-DCouAgm)	20 μM (12.2 μg/mL)^34^
*Bipolaris sorokiniana*	FerAgm	no activity^22^
	FerAgm-2-7′/8-8′-DFerAgm	1 mM (610 μg/mL)^22^
	FerAgm-8-8′/9-*N*-7′-DFerAgm	1 mM (610 μg/mL)^22^
*Fusarium asiaticum*	FerAgm	no activity^22^
	FerAgm-2-7′/8-8′-DFerAgm	no activity^22^
	FerAgm-8-8′/9-*N*-7′-DFerAgm	0.3 mM (183 μg/mL)^22^
*Erypsiphe graminis* (powdery mildew)	CouAgm	72 mM (20 mg/mL)^35^
	CouAgh	68 mM (20 mg/mL)^35^

a The systematic nomenclature of van Zadelhoff et al. (2022)^21^ is used in this work. Agm = agmatine, Agh = hydroxyagmatine.

In plants, (neo)­lignanamides are formed via oxidative coupling of phenolamides, which results in a heterogeneous class of compounds^21^ that are also present in barley, barley malts, beer, and brewing byproducts.^24,25^ The dimers of HCAgms, commonly referred to as hordatines, have been reported to possess potent antimold activity as inhibitors of the germination of fungal spores at concentrations of 5 to 10 μg/mL.^26^ Although HCAgms have been detected in various plants, such as wheat and rice,^27,28^ hordatines are produced exclusively in wild and cultivated barley (*Hordeum* spp.)^22^ as their biosynthesis is proposed to be catalyzed by specific barley laccases whose regio- and stereoselectivity is steered by cofactors called dirigent proteins.^29,30^ The two main compounds of this class are hordatine A (CouAgm-4-*O*-7′/3–8′-DCouAgm), the dimer of *p*-coumaroylagmatine, and hordatine B (FerAgm-4-*O*-7′/3–8′-DCouAgm), the heterodimer of *p*-coumaroylagmatine and feruloylagmatine.^31,32^ Both these hordatines are described as *trans*-isomers with *C*-7′*S* and *C*-8′*S* absolute configuration.^16,29,33^

Hordatines are just few of the dimers of hydroxycinnamic acids or their derivatives that are formed via oxidative coupling.^36^ Another of such dimers is poacic acid, a 3–8′ dimer of ferulic acid and decarboxylated ferulic acid.^37^ Poacic acid is present in various grasses (*Poaceae*), such as maize and brans and grains of other cereals,^38,39^ and in cell walls of barley leaves.^40^ Its fungicidal activity has been reported against a plethora of yeast and mold strains, such as *S. cerevisiae*,^37^ and the oomycete pathogens *Sclerotinia sclerotiorum, Alternaria solani, and Phytophthora sojae*.^37^ Thus, poacic acid is a multitarget antifungal (i.e., antiyeast and antimold) compound and possesses a complex mechanism of action that is not only attributed to its interaction with β-1,3-glucan within fungal cell walls,^37^ but also to the disruption of metal homeostasis and chitin production.^41^

Barley-derived beer brewing byproducts (e.g., barley rootlets) contain a wide diversity of metabolites, including HCAgms and their dimers,^25^ many of which have not yet been evaluated for their antiyeast potential. One of the challenges in this respect is that the purification and isolation process of these compounds from complex barley extracts is time-consuming, cost-intensive, and low yielding. Consequently, their complete biological evaluation is troublesome. Therefore, the main aims of this study were (i) to obtain dimers of hydroxycinnamic acids and their derivatives and (ii) to assess their activity against the beer spoilage yeast *S. cerevisiae* subsp. *diastaticus*.

It was previously demonstrated that the oxidative coupling reaction responsible for dimerization of barley HCAgms can be mimicked in vitro using the enzyme horseradish peroxidase (HRP) in the presence of H_2_O_2_.^42,43^ This study builds on these findings and aims to develop a chemo-enzymatic synthetic strategy to obtain dimers of ferulic acid, including poacic acid, and feruloylagmatine (FerAgm), including *rac*-hordatine A. The resulting structures included dimers naturally occurring in barley, as well as structural analogues of those compounds that were used to gain insights into the effect of various structural features on their antimicrobial activity. The dimers were evaluated for their activity against the wild yeast *S. cerevisiae* subsp. *diastaticus*. Subsequently, flash chromatographic separation of a barley rootlet extract was used to obtain pools of natural HCAgm dimers with various natural modifications (e.g., glycosylation). Since the standardized media that are typically used for antiyeast assays are not representative for the food product of interest, i.e. beer, these pools were also tested for their antiyeast activity in media produced from alcohol-free beer and regular beer.

## 2 Material and Methods

### 2.1 Materials

#### 2.1.1 Chemicals

All reagents and solvents were purchased from commercial suppliers and used without further purification. HPLC-grade acetonitrile (ACN), ULC–MS grade ACN and water, formic acid (FA) (99% [*v*/*v*]), ethanol (EtOH), and methanol (MeOH) were purchased from Biosolve (Valkenswaard, The Netherlands). Nisin, WL nutrient agar, malt extract agar (MEA), and potassium sorbate were purchased from Merck (Darmstadt, Germany). Water for purposes other than UHPLC was purified using a Milli-Q purification system equipped with a 0.22 μm filter (Merck Millipore, Molsheim, France).

#### 2.1.2 Biological Materials

Peptone physiological saline (PPS) was bought from Tritium Microbiologie (Eindhoven, The Netherlands). Bacteriological agar was bought from VWR International (Leuven, Belgium). De Man, Rogosa, Sharpe (MRS) broth and MRS agar were purchased from Oxoid LTD (Basingstoke, Hampshire, England). Yeast extract peptone dextrose (YPD) broth was bought from Scharlab S.L. (Barcelona, Spain). All other chemicals used in this research were of analytical grade. *Aspergillus niger* CBS 114.39 (19) and *Penicilium commune* CBS 468.84 from Westerdijk Fungal Biodiversity Institute (Utrecht, The Netherlands). Microbial strains isolated from the brewery, being *L. brevis* MB124 and *S. cerevisiae* subsp. *diastaticus* MB523, were provided by Heineken (Zoeterwoude, The Netherlands) and were stored at – 80 °C in glycerol stocks before usage. The tested strain of *S. cerevisiae* subsp. *diastaticus* was an industrial isolate from Heineken’s collection, thus this strain has been extensively exposed to beer. Barley (*Hordeum vulgare*) rootlets from malt produced in Portugal were supplied by Heineken (Zoeterwoude, The Netherlands).

### 2.2 Methods

#### 2.2.1 Extraction of HCAgm Dimers and Their Derivatives from Barley Rootlets

Barley rootlets (50 g) were defatted with hexane (2 cycles, 20 mL/g/cycle material, 20 min sonication/cycle), followed by hexane removal through filtration. The retentate was dried, followed by a methanol extraction (4 cycles, 20 mL/g/cycle material, 20 min sonication/cycle). Solids were removed by filtration and the permeate was analyzed by reversed-phase ultrahigh performance liquid chromatography combined with photodiode array detection and ion trap mass spectrometry (RP-UHPLC-PDA-IT-MS^*n*^) (Section 2.2.8) to check for the presence of the compounds of interest. The methanol was removed under reduced pressure and the obtained material was used for fractionation by flash chromatography.

#### 2.2.2 Fractionation of a Barley Rootlet Extract by Flash Chromatography

The dried methanol extract was dissolved in a 5% (*v*/*v*) ACN solution (7.5 mL/g of extract) and sonicated for 15 min. The extract was centrifuged at 10,000*g* for 5 min and the supernatant was collected to be used for the flash chromatography. The extract solutions were manually injected onto a 40 g Reveleris C18 RP column. Water (A) and ACN (B), both acidified with 1% (*v*/*v*) FA, were used as eluents. The column was equilibrated with 5 column volumes (CV) of eluent A. The following elution profile was used: 0–2 CV, linear gradient to 5% B; followed by 2–22 CV, linear gradient to 10% B; 22–32 CV, linear gradient to 20% B; 32–33 CV, linear gradient to 100% B; 33–35 CV, isocratic at 100% B; 35–37 CV, linear gradient to 5% B. UV signal was detected at 280 and 320 nm. The fractions collected were first analyzed by RP-UHPLC-PDA-IT-MS^*n*^ and then pooled based on similarity of their composition. The composition of the barley rootlet dimer pools was determined by RP-UHPLC-PDA-IT-MS^*n*^. The solvent was evaporated under reduced pressure followed by freeze-drying.

#### 2.2.3 Synthesis and Purification of Hydroxycinnamic Acid and Hydroxycinnamoylagmatine (HCAgm) Dimers

Three hydroxycinnamoylagmatines (HCAgms), namely *p*-coumaroylagmatine (CouAgm), feruloylagmatine (FerAgm), and sinapoylagmatine (SinAgm), were synthesized as described in van Zadelhoff et al. (2022).^20^ The synthesis of HCAgm dimers was achieved using two different approaches both involving horseradish peroxidase (HRP) catalyzed oxidative coupling. Starting from HCAgms, **4**–**10** were synthesized, purified and identified as described in van Zadelhoff et al. (2022).^42^ Compounds **12**, **13**, **14**, and **15** (poacic acid) were synthesized starting from ferulic acid and ethyl ferulate, after which these hydroxycinnamic acid dimers were purified, and identified (Scheme 1). Subsequently, they were further functionalized with agmatine to give HCAgm dimers **5** and **11** (Scheme 2).^42^

**Scheme 1:**
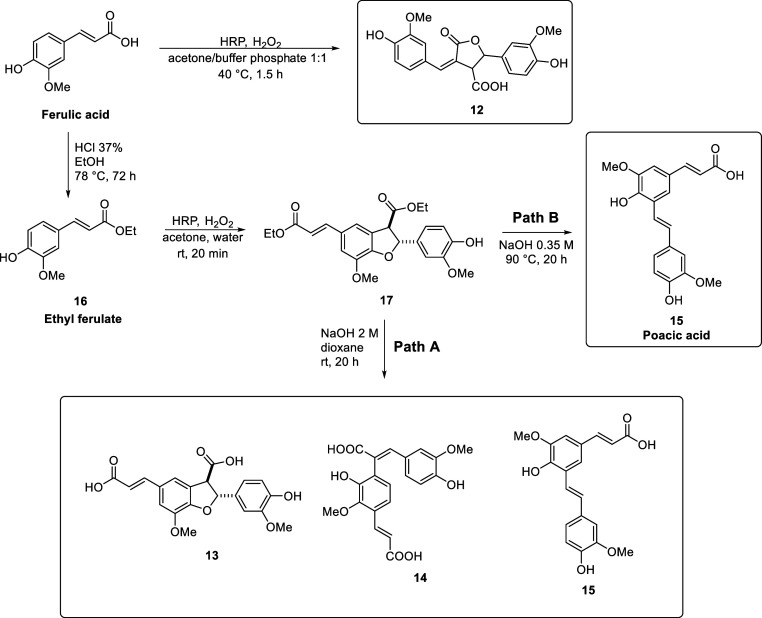
Synthesis of Ferulic Acid Dimers via HRP/H_2_O_2_ Oxidative Coupling Starting from Ferulic Acid and Ethyl ferulate (**16**)

**Scheme 2:**
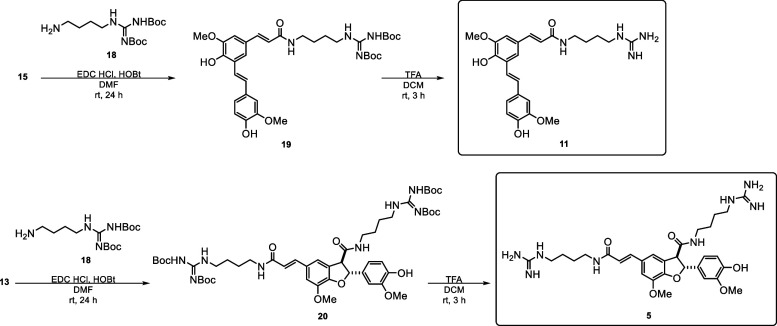
Synthesis of HCAgm Dimers **5** and **11** by Amidation of Ferulic Acid Dimers **13** and **15** with Agmatine

All ^1^H NMR and ^13^C NMR spectra were recorded with a Bruker AV600 (^1^H, 600 MHz; ^13^C, 150 MHz) or a Bruker AMX-300 (^1^H, 300 MHz; ^13^C 75 MHz) spectrometer. Chemical shifts (δ) are expressed in ppm, and coupling constants (J) are in Hz. NMR spectral data of all compounds are presented in the Supporting Information. High resolution ESI-MS data were obtained using a Synapt G2-Si Q-ToF mass spectrometer (Waters Corporation, Wilmslow, UK) with a ZSpray ESI-probe (Waters Corporation). All reactions requiring anhydrous conditions were performed under a positive nitrogen flow, and all glassware was oven-dried. Isolation and purification of the compounds were performed by flash column chromatography on silica gel 60 (230–400 mesh). Thin layer chromatography (TLC) analyses were performed using commercial silica gel 60 F254 aluminum sheets.

##### 2.2.3.1 Synthesis of Compound **12**

Ferulic acid (78 mg, 0.4 mmol, 1 equiv) and HRP (160 μL, 1 mg/mL in water) were suspended in a 1:1 solution of acetone/buffer phosphate (pH = 8) (2 mL, [0.2 M]) and heated at 40 °C. After 30 min, a solution of H_2_O_2_ (30% [*w*/*w*]) was added (60 μL, 1.47 equiv), the reaction was stirred for an additional 1.5 h, and then cooled at room temperature. The crude reaction mixture was extracted with ethyl acetate, and the combined organic layers were dried over Na_2_SO_4_, filtered, and evaporated under reduced pressure. Purification by column chromatography (hexane/ethyl acetate 1:9 with 0.5% acetic acid) gave the desired product **12**, Fer-8-8′/9-*O*-7′-Dfer, as a brown solid in 25% yield.

##### 2.2.3.2 Synthesis of Compounds **13**, **14**, and **15**

To a suspension of ferulic acid (6 g, 30.8 mmol, 1 equiv) in ethanol (86 mL) was added 30 drops of HCl 37% (*w*/*w*). The reaction was refluxed at 78 °C for 72 h, then concentrated under reduced pressure. The crude was solubilized in ethyl acetate and washed with a saturated solution of NaHCO_3_ and a saturated solution of NaCl. The organic layer was dried over Na_2_SO_4_, filtered, and evaporated under reduced pressure. The desired product **16** was obtained as a brown oil in 98% yield and was used in the next step without further purification.

Ethyl ferulate **16** (2.52 g, 11.34 mmol, 1 equiv) was dissolved in acetone (0.22 M) and diluted with water (148.50 mL) to obtain a -milky solution. H_2_O_2_ (30% [*w*/*w*]; 0.63 mL, 6.2 mmol, 0.5 equiv) and a solution of HRP in water (1.14 mg, 0.40 mg/mL) were sequentially added. The reaction mixture was further diluted with H_2_O (86 mL) and stirred vigorously at room temperature for 20 min, then acidified with HCl (pH < 3) and extracted with ethyl acetate. The organic phase was washed with a saturated solution of NaCl, dried over Na_2_SO_4_, filtered, and concentrated under reduced pressure. Purification by column chromatography (cyclohexane/ethyl acetate 75:25) gave the desired product **17** as a white solid in 22% yield.

##### 2.2.3.6 Path A: Synthesis of the Dimeric Compounds **13**, **14**, and **15**

The ethyl ferulate dimer **17** (540 mg, 1.2 mmol, 1 equiv) was dissolved in dioxane dry (12.2 mL) and cooled at 0 °C under a N_2_ atmosphere. A degassed solution of NaOH (2 M, 30 mL, 48 equiv) was added, and the reaction was allowed to reach room temperature. After 20 h, the reaction mixture was acidified with HCl 37% (*w*/*v*; pH = 1) and extracted with ethyl acetate. The organic phase was washed with a saturated solution of NaCl, then dried over anhydrous Na_2_SO_4_, filtered, and evaporated under reduced pressure. Purification by column chromatography (toluene/ethyl acetate/isopropyl alcohol 65:30:5 with 0.5% acetic acid) gave a mixture of the desired products **13**, **14**, and **15**.

(**13**) Fer-4-*O*-7′/3-8′-DFer, white solid, 38% yield.

(**14**) Fer-8-5′-DFer, white solid, 35% yield.

(**15**) Poacic acid (Fer-3-8′-decarboxyFer), yellow solid, 16% yield.

##### 2.2.3.7 Path B: Synthesis of Compound **15**

The ethyl ferulate dimer **17** (400 mg, 0.9 mmol, 1 equiv) was suspended in a solution (0.35 M) of NaOH (14.4 mL) under N_2_ atmosphere and stirred at 90 °C for 20 h. The reaction was cooled to 10 °C, acidified with HCl 37% (*w*/*w*; pH = 1), and extracted with ethyl acetate. The organic phase was washed with a saturated solution of NaCl, dried over anhydrous Na_2_SO_4_, filtered, and evaporated under reduced pressure. Purification by column chromatography (ethyl acetate/ethanol/hexane 36:4:40 with 0.4% acetic acid) gave the desired product **15**, poacic acid (Fer-3-8′-decarboxyFer), as a yellow solid in 63% yield.

##### 2.2.3.8 General Procedure for the Agmatine Functionalized Compounds **19** and **20**

A dichloromethane solution (15 mL) of 1,3-di-Boc-2-(trifluoromethylsulfonyl)­guanidine (325 mg, 0.83 mmol, 0.3 equiv) was added dropwise to a solution of putrescine (243 mg, 2.76 mmol, 1 equiv) and triethylamine (84 mg, 0.83 mmol, 0.3 equiv) in anhydrous dichloromethane (38 mL). The reaction mixture was stirred at room temperature for 14 h, then the solvent was evaporated under reduced pressure. Purification by column chromatography (dichloromethane/methanol 98:2 to dichloromethane/methanol/triethylamine 100:3:2) gave the desired product **18** as a white solid in quantitative yield.

EDC HCl (1.5 equiv) and HOBt (1.5 equiv) were added to a solution of the selected acid (1 equiv) and bis-Boc protected agmatine **18** (1.5 equiv) in anhydrous DMF (0.05 M). The reaction mixture was stirred at room temperature for 24 h, diluted with ethyl acetate, and washed three times with a saturated solution of NaCl. The organic layer was dried over Na_2_SO_4_, filtered, and concentrated under reduced pressure. Purification by column chromatography (dichloromethane/methanol 100:1) gave the desired product.

(**19**) Purification by column chromatography dichloromethane/methanol 100:1 afforded the desired product **19** as a pale yellow solid in 60% yield.

(**20**) Purification by column chromatography with a gradient of cyclohexane/ethyl acetate 3:7 to 1:9 afforded the desired product **20** as a white solid in 46% yield.

##### 2.2.3.9 General Procedure for the Boc Deprotection to Give Compounds **5** and **11**

The selected Boc-protected derivative (1 equiv) was solubilized in dichloromethane (0.10 M) and cooled at 0 °C. TFA (10 eq for each Boc group) was added dropwise, and the reaction was allowed to reach room temperature. After 3 h, the solvent was evaporated under reduced pressure.

(**5**) Trituration with diethyl ether gave the desired product **5** as a white solid in quantitative yield.

(**11**) Purification by column chromatography with a gradient of dichlorometane/methanol 9:1 to 8:2 gave the desired product **11** as a pale yellow solid in 39% yield. High resolution ESI-MS *m*/*z* 455.2291 [M + H]^+^ (calcd for C_24_H_30_N_4_O_5_, 454.2216).

The structures of all compounds investigated in this work are shown in Figure 1.

**Figure 1:**
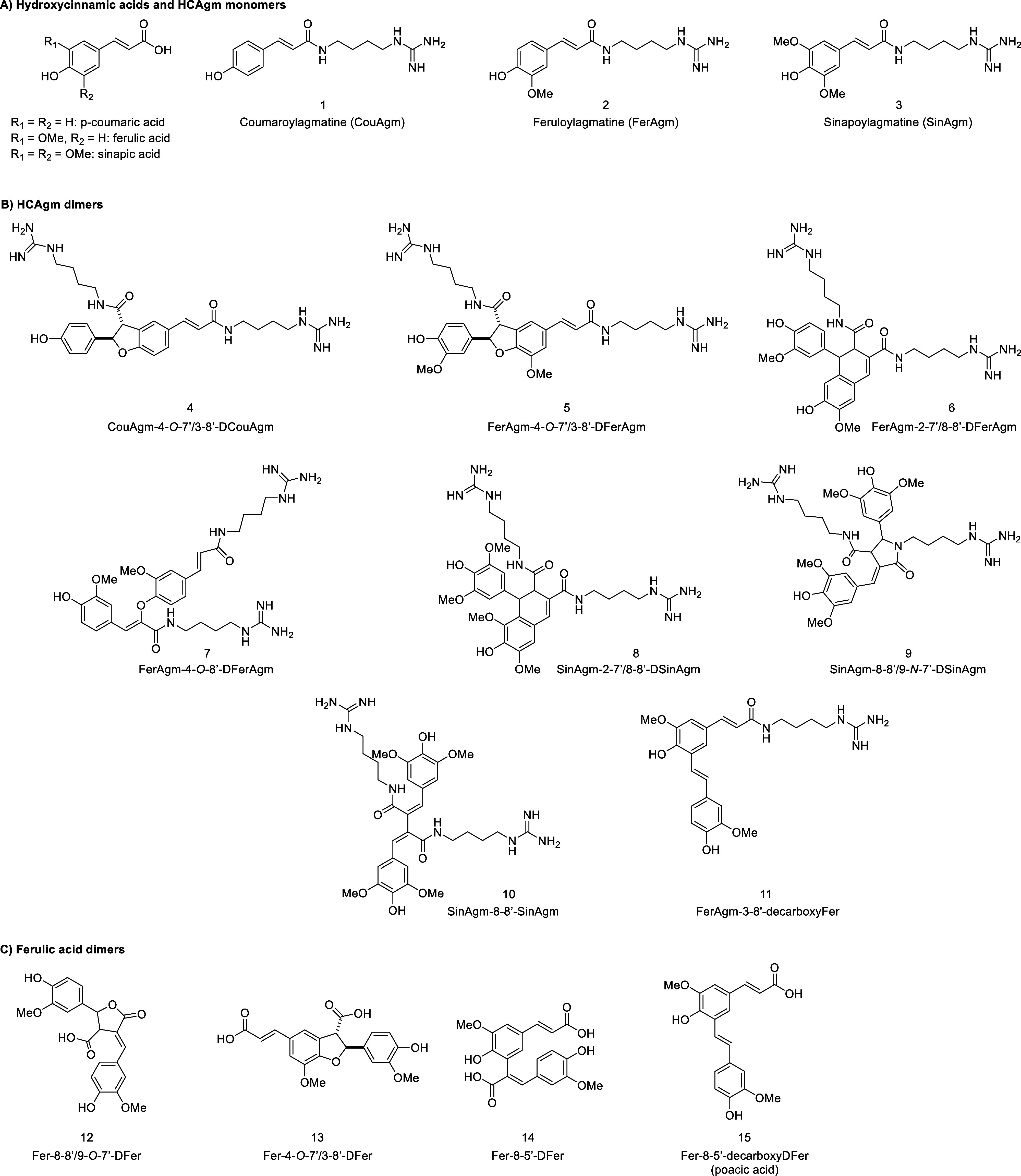
Structures of hydroxycinnamic acids and hydroxycinnamoylagmatine (HCAgm) monomers (A), HCAgm dimers (B), and ferulic acid dimers (C) investigated in this work.

#### 2.2.4 Preparation of Stock Solutions and Growth Media

The HCAgms, HCAgm dimers, and barley rootlet dimer pools were dissolved in 60% (*v*/*v*) ethanol to prepare sterile stock solution with a concentration of 25,000 μg/mL. These stock solutions were stored at – 20 °C until use.

As standardized media, YPD broth was used for *S. cerevisiae* subsp. *diastaticus* and MRS broth for *L. brevis*. The pH of both broths was adjusted to pH 4.4 by addition of 1 M HCl to mimic the acidic environment of beer.^44^ After adjustment, a 0.2 μm filter was used to sterilize the broth. The broths at their default pH, i.e. pH 6.5, were also used to compare the impact of pH on the antimicrobial activity.

To prepare the beers for antimicrobial testing, both alcohol-free (Heineken Pilsener 0.0) and traditional beer (Heineken Pilsener Original) were first degassed using an ultrasonic bath and, prior to adding the target microorganism, the beers were sterilized through a 0.45 μm filter to obtain the beer broths. For the beer agars, the sterilized beers were heated to 50 °C and mixed with a 3.5% (*w*/*v*) autoclaved bacteriological agar solution in a 2:1 volume ratio. After thorough mixing, the mixture was poured into plates to solidify.

#### 2.2.5 Antiyeast Activity Assays in Standardized Media

The antiyeast activity of HCAgms, barley rootlet dimer pools, and compounds **4**–**15** was tested using the broth microdilution assay. In short, the yeast was precultured for 24 h at 30 °C on a plate of agar. After preculturing, one colony of the yeast was transferred to the broth which was subsequently incubated for 18 h at 30 °C. The obtained culture was diluted in broth to obtain the initial inoculum of 5 log_10_ colony forming units (CFU) per mL. Stock solutions of the compounds of interest were made in 60% (*v*/*v*) EtOH. These stock solutions were diluted to the concentrations to be tested using broth. The highest final concentration of EtOH was 3.2% (*v*/*v*), which did not affect yeast growth (data not shown). The highest dimer concentration tested was 250 μg/mL and for monomers this was 500 μg/mL. A 96-well plate was filled, with in each well 100 μL of one of the compounds of interest and 100 μL of initial inoculum. Broth with 2% (*v*/*v*) EtOH was used as a negative control. Potassium sorbate at 3 mg/mL was used as a positive control in the antiyeast assay. Additionally, the compounds of interest at their lowest concentrations and broth without yeast were used to check for contaminations. The 96-well plate was sealed with a moisture barrier film (Azenta, Burlington, United States) and the plate was incubated at 30 °C in an Infinite 200 Pro M nanoplate reader (Tecan, Männedorf, Switzerland) for 24 h. The optical density (OD) at 600 nm was read every 5 min after shaking for 15 s. Time to detection (TTD), an indicator of yeast growth inhibition, was defined as the time required to achieve a 0.05 change in OD_600_.^45^ After 24 h of incubation, cell viability was verified by plate counting for wells in which no change in OD or a change in the growth curve in comparison with the negative control was observed. For this, 100 μL of the corresponding well was serially diluted in PPS solution and 100 μL of the solution was spread on a plate of the respective agar. After an incubation of 48 h at 30 °C, the colonies on the agar plate were counted (i.e., end count ≤5 log CFU/mL). This was determined by cell viability determination by plate counting for wells where no increase in OD was observed. The MIC was determined as the concentration at which the CFU/mL was the same or lower than that of the initial inoculum with a maximum accepted increase of 0.3 log_10_ CFU/mL. The minimum bactericidal concentration (MBC) was defined as the lowest concentration that resulted in >99.9% (i.e., 3 log reduction) inactivation from the initial inoculum. All experiments were performed in technical duplicate, and active compounds were additionally tested in biological triplicates.

#### 2.2.6 Antiyeast Activity Assays in Beer

The potential for wild yeast inhibition was determined in alcohol-free (Heineken 0.0%) and regular beer (Heineken pilsener) were obtained from a local supermarket. Only two beers were selected, as the main goal was to perform an initial exploration of the antiyeast potential of the compounds in a beer matrix. The method described in Section 2.2.5 was used with some adjustments. Yeast was grown on beer agar plates and beer broth was used as for all liquid cultures. In real-life applications, beer would already be severely spoiled at 5 log_10_ CFU/mL. Therefore, an initial inoculum of 1 log_10_ CFU/mL was used to mimic a realistic initial level of contamination of beer. Due to this lower initial inoculum size, no increase in optical density could be measured spectrophotometrically, because the OD_600_ was below the detection limit of the nanoplate reader. Therefore, no TTD could be determined in these experiments. Consequently, after 24 h incubation in the nanoplate reader, all wells were subjected to cell viability verification via plate counting. For this purpose, WL nutrient agar plates were used on which 100 μL of either the content of the well or 100 μL of a serial dilution was spread. The MIC was determined as the concentration at which the CFU/mL was the same or lower than that of the initial inoculum with a maximum accepted increase of 0.3 log_10_ CFU/mL. All experiments were performed in biological triplicates, in technical triplicates. Active compounds were additionally tested in biological duplicates.

#### 2.2.7 Antibacterial and Antimold Activity Assays in Standardized Media

The potentially broader scope of the antimicrobial activity of the compounds was investigated by testing against the bacterium *L. brevis* and two molds. *L. brevis* was used to assess the antibacterial activity of the compounds and was selected because it is a known beer spoilage organism.^8,9^ To assess the antimold activity and allow comparison with previous reports on the antimold activity of hordatines, the two molds *A. niger* and *Penicillium commune* were used, since *Aspergillus* and *Penicillium* were reported to cause spoilage during barley storage and malting.^7,8^

To test HCAgms and barley rootlet dimer pools for their antibacterial activity against *L. brevis*, a method identical to the antiyeast activity assays in standardized media (see Section 2.2.5) was used with the following adaptation: Preculturing was performed for 48 h. Similar to the tested yeast strain, the tested strain of *L. brevis* was an industrial isolate from the Heineken collection.

For the antimold activity against multicellular fungi, the disc diffusion assay was performed at a concentration of 100 μg/mL and 10 mg/mL. The two molds, *A. niger* and *P. commune*, were grown on malt extract agar plates. After 24 h of incubation, the spores were removed with a loop and were transferred to a slant with malt extract agar. After they were fully grown, PPS solution was added and a loop was used to release the spores. The spore containing solution was spread on agar plates with discs wetted with 10 μL of the compound of interest solution or a positive control were used. The antimold activity could then be determined by the inhibition zone.

#### 2.2.8 RP-UHPLC-PDA-IT-MS^*n*^ Analysis

To determine the composition of the barley rootlet dimer pools and the beers that were used to produce media for the microbiological experiments, RP-UHPLC-PDA-IT-MS^*n*^ was used. The samples were separated on a Thermo Vanquish UHPLC system (Thermo Scientific, San Jose, CA) equipped with a pump, degasser, autosampler, and PDA detector. Samples (1 μL) were injected on a Waters Acquity BEH C18 column (150 mm × 2.1 mm i.d., 1.7 μm particle size) with a VanGuard guard column of the same material (5 mm × 2.1 mm i.d., 1.7 μm particle size) (Waters, Milford, MA, USA). The flow rate was set to 400 μL/min at a column temperature of 35 °C. The PDA detector was set to measure wavelengths in the range of 190–680 nm. Water (A) and ACN (B), both acidified with 1% (*v*/*v*) formic acid, were used as eluents. The following elution profile was used: isocratic at 0% B for 1.09 min; 1.09–45.05 min, linear gradient to 20% B; 45.05–46.51 min, linear gradient to 100% B; and 46.51–53.77 min, isocratic at 100% B. The eluent was adjusted to its starting composition in 1.10 min followed by equilibration for 5.49 min.

Mass spectrometric data were acquired using a Velos Pro linear ion trap mass spectrometer (Thermo Scientific, San Jose, CA, USA) equipped with a heated ESI probe coupled inline to the Vanquish RP-UHPLC system. Nitrogen was used as sheath gas (50 arbitrary units) and auxiliary gas (13 arbitrary units). The source conditions were a capillary temperature of 263 °C, a probe heater temperature of 425 °C, and a source voltage of 3.5 kV. The S-Lens RF level was 67.63%. Data was collected in positive ionization mode over the *m*/*z* range 250–1500. Fragmentation of the most abundant ions in full MS was performed by collision-induced dissociation (CID) with a normalized collision energy of 35%. Dynamic exclusion with a repeat count of 3, a repeat duration of 5.0 s, and an exclusion duration of 10.0 s was used to obtain MS^2^ spectra of multiple different ions present in full MS at the same time. Most settings were optimized via automatic tuning using LTQ Tune Plus (Xcalibur version 4.1, Thermo Scientific). Data processing was performed using Xcalibur 4.1 (Thermo Scientific).

## 3 Results and Discussion

### 3.1 Chemo-Enzymatic Synthesis of Ferulic Acid and Feruloylagmatine Dimers

Naturally occurring dimers of ferulic acid, e.g. poacic acid, and feruloylagmatine (FerAgm), e.g. hordatine C, were previously reported to possess antimicrobial properties.^26,37^ Therefore, ferulic acid and FerAgm dimers have been synthesized following a combination of previously reported methods and their antiyeast activity have been investigated.^36,46,47^ The synthetic pathway followed is reported in Schemes 1 and 2. In detail, subjecting ferulic acid to HRP/H_2_O_2_ oxidative coupling in a 1:1 acetone/phosphate buffer (pH 8) at 40 °C yielded Fer-8-8′/9-*O*-7′-DFer (**12**) as a major product (Scheme 1). Alternatively, **17** was isolated when ethyl ferulate was suspended in acetone/water solution and subjected to HRP/H_2_O_2_ oxidative coupling. This key intermediate opened the possibilities to obtain in hydrolytic conditions the diacid (**13**), a precursor of FerAgm-4-*O*-7′/3-8′-DferAgm (**5**). It is worth mentioning that dimerization reactions occur with regio- and diastereoselectivity, but not enantioselectivity, affording racemic mixtures of the *trans*-isomer.^48^

Additionally, **17** was exploited as an intermediate to access other ferulic acid dimers by controlling the hydrolytic conditions. When compound **17** was treated with 2 M NaOH at room temperature (Scheme 1, Path A) compounds **13**, **14**, and **15** were obtained and separated by column chromatography in 38%, 35%, and 16% yield, respectively. When the hydrolysis was carried out in a diluted system (0.35 M NaOH) at 90 °C, poacic acid (**15**) was the only isolated product in 63% yield. This is particularly relevant synthetic route, since poacic acid (**15**) is known to be endowed with significant antifungal properties.^37^

Further functionalization of **13** via EDC HCl and HOBt with protected agmatine (**18**) afforded **20**, and after deprotection in TFA/DCM, the desired compound **5** (Scheme 2).

Compared to the previously reported route via HRP/H_2_O_2_ oxidative coupling of FerAgm (**2**) followed by flash chromatography and preparative HPLC purification,^42^ a major advantage of the route described here is that no further chromatographic purification is required, thereby making it much less laborious. The same functionalization with protected agmatine (**18**) was applied to compounds **12**, **14**, and **15**. However, the formation of amides from **12** and **14** was not successful, most likely because of the equilibrium between closed lactones and open hydroxy acids. On the other hand, amidation of poacic acid (**15**) gave compound **11** in high yield (Scheme 2).

### 3.2 Oxidative Coupling and Amidation can Enhance the Antiyeast Activity of Ferulic Acid Derivatives

Poacic acid (Fer-3-8′-decarboxyFer; **15**), three additional ferulic acid dimers (**12**–**14**), a poacic acid-derived agmatine amide (FerAgm-3-8′-decarboxyFer; **11**), and *rac*-hordatine C (FerAgm-4-*O*-7′/3-8′-DFerAgm; **5**) were tested against *S. cerevisiae* subsp. *diastaticus* to explore their antiyeast activity and gain insights into the structure–activity relationships of ferulic acid and FerAgm dimers. The compounds’ MIC and MBC values against this wild yeast are provided in Table 2.

**Table 2:** Minimum Inhibitory Concentration (MIC) and Minimum Bactericidal Concentration (MBC) in μg/mL and μM of Ferulic Acid, Ferulic Acid Dimers, and Their Agmatine Amides against *S. cerevisiae* subsp. *diastaticus* ^b^

	MIC		MBC	
compound	μg/mL (LR^a^)	μM	μg/mL (LR)	μM
ferulic acid	>1000 (n.a.)	>5150	>1000 (n.a.)	>5150
agmatine	>1000 (n.a.)	>7681	>1000 (n.a.)	>7681
feruloylagmatine (FerAgm; **2**)	>1000 (n.a.)	>3264	>1000 (n.a.)	>3264
*rac*-hordatine C (FerAgm-4-*O*-7’/3-8′-DFerAgm; **5**)	1000 (2.11 ± 2.68)	1637	>1000 (n.a.)	>1637
Fer-4-*O*-7′/3-8′-DFer (**13**)	>1000 (n.a.)	>2588	>1000 (n.a.)	>2588
Fer-8-8′/9-*O*-7′-DFer (**12**)	>1000 (n.a.)	>2588	>1000 (n.a.)	>2588
Fer-8-5′-DFer (**14**)	>1000 (n.a.)	>2588	>1000 (n.a.)	>2588
poacic acid (Fer-3-8′-decarboxyFer; **15**)	>1000 (n.a.)	>2921	>1000 (n.a.)	>2921
FerAgm-3-8′-decarboxyFer (**11**)	500–750 (3.81 ± 1.91)	1100–1650	500–750 (3.81 ± 1.91)	1100–1650
traditional food preservative (potassium sorbate)	20,000–25,000 (1.24 ± 0.49)	178,364–222,956	30,000 (3.01 ± 0.13)	267,547

a LR = log reduction; n.a. = not applicable.

b Compound numbers refer to Figure 1. The traditional food preservative potassium sorbate was included as positive control. Compounds were tested at 5 log_10_ CFU/mL in YPD broth at pH 6.5.

At the inoculum size used in this experiment, no inhibition of *S. cerevisiae* subsp. *diastaticus* was observed at concentrations up to 1000 μg/mL for ferulic acid, agmatine, FerAgm (**2**), or any of the ferulic acid dimers, including poacic acid (**15**). This difference compared to the previous report by Piotrowski et al. (2015) is possibly due to differences in the initial inoculum or strain, although they do not provide this detailed information in their study.^37^ In particular, the yeast strain used in this study was isolated from a brewery and could, therefore, already have gained resistance to these compounds upon extensive exposure to natural ferulic acid derivatives from beer. The two compounds that inhibited the growth of the yeast were FerAgm-4-*O*-7′/3-8′-DFerAgm (**5**) and FerAgm-3-8′-decarboxyFer (**11**). For **11**, which possesses a stilbenoid backbone, an MBC could even be established at 500–750 μg/mL (1100–1650 μM). These two active compounds are the agmatine amides derived from the inactive ferulic acid dimers **13** and **15**, respectively. Besides this, it is evident from the comparison of FerAgm (**2**) and FerAgm-4-*O*-7′/3-8′-DFerAgm (**5**) that oxidative coupling can enhance antiyeast activity.

Despite the apparent discrepancy in activity between what was observed for the ferulic acid dimers in this study and the report by Piotrowski et al. (2015),^37^ the results presented in this study regarding the two most potent linkage types, i.e. 4-*O*-7′/3-8′ and 3-8′, are in line with their observations. Ferulic acid dimers and amides have been investigated as lead compounds for the development of novel crop protection or antiviral agents, demonstrating that there is a broader spectrum of potential applications for these compounds.^23,49^ Overall, the results presented in this work indicate that functionalization with agmatine, as well as oxidative coupling, can be tools to increase the antiyeast activity of hydroxycinnamic acid derivatives.

### 3.3 Antiyeast Potential of Natural Phenolamides from Barley Rootlets

Considering the promising antiyeast activity of the two phenolamides **5** and **13** obtained via chemo-enzymatic synthesis, the aim was to explore whether phenolamides from barley rootlets, a beer brewing byproduct, can potentially be used as preservatives for alcohol-free beer. Barley rootlets were previously shown to contain a wide diversity of hydroxycinnamoylagmatines (HCAgms), including FerAgm dimers.^25^

#### 3.3.1 Isolation and Characterization of Barley Rootlet Dimer Pools

The content of the hydroxycinnamoylagmatines (HCAgms) and related compounds in barley rootlet extracts was only between 1.5% and 4.0% (quantified based on a calibration curve of purified dimer **4**, data not shown). To gain more insight into the potency of these compounds and the effect of various structural features, four barley rootlet dimer pools enriched in HCAgm derivatives were produced for antiyeast testing. For industrial applications, fractionation of extracts is expected to be too expensive, however, testing these pools can give valuable information about the activity of specific compounds within the extract. Pools were obtained by flash fractionation of a barley rootlet extract. Characterization of the HCAgm derivatives in the pools was based on the guideline described in van Zadelhoff et al. (2024).^25^ The chromatographic profiles of these four pools (Figure 2A–D) revealed that they predominantly consisted of both hydroxylated and glycosylated dimers (**A**), glycosylated dimers (**B**), hydroxylated dimers (**C**), and nonsubstituted dimers (**D**). Based on the UV_320 nm_ chromatograms and MS chromatograms, the pools were sufficiently enriched in the compounds of interest.

**Figure 2:**
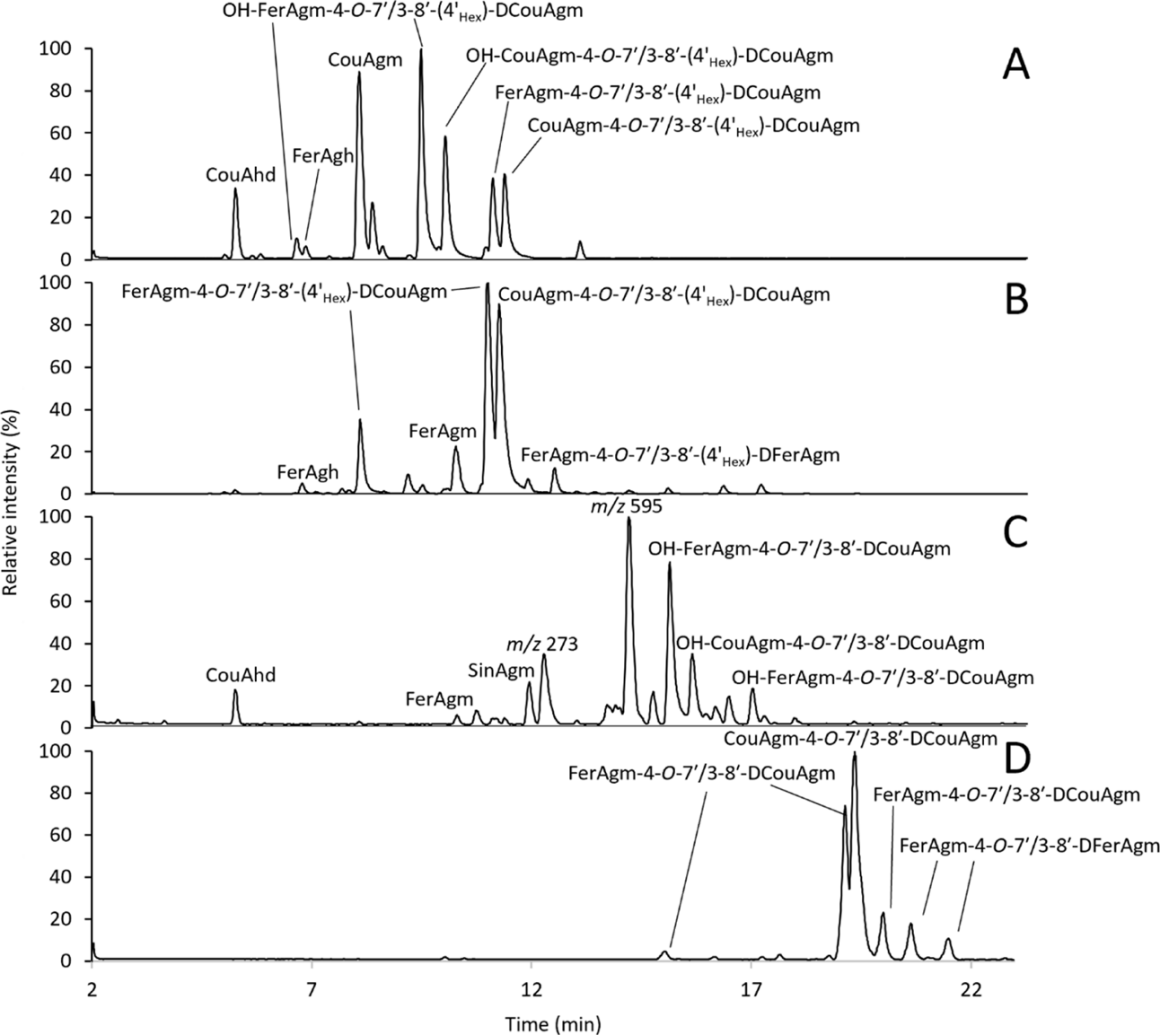
RP-UHPLC-IT-MS base peak chromatograms (*m*/*z* 200–1500) in positive ionization mode for the hydroxylated and glycosylated dimer pool (**A**), the glycosylated dimer pool (**B**), the hydroxylated dimer pool (**C**), and the nonsubstituted dimer pool (**D**) from the barley extract. Pool C contains two unknown compounds: *m*/*z* 273, an unknown agmatine-related compound; and *m*/*z* 595, an unknown nonagmatine-related compound.

The pools contained derivatives of FerAgm-4-*O*-7′/3-8′-DFerAgm (**5**), which was one of the active compounds based on the previous experiment (Table 2). Besides this, the pools primarily consisted of other 4-*O*-7′/3-8′-linked HCAgm dimers, such as derivatives of CouAgm-4-*O*-7′/3-8′-DCouAgm (**4**) and FerAgm-4-*O*-7′/3-8′-DCouAgm. None of the other compounds obtained via chemo-enzymatic synthesis, like poacic acid (**15**) or FerAgm-3-8′-decarboxyFer (**11**), were detected in the pools.

#### 3.3.2 Identification and Quantification of HCAgms and Their Dimers in Beer

Beer was reported to contain HCAgms and dimers thereof.^24,50^ Therefore, before testing the potency of the monomers and dimers as natural preservatives in beer, the variety and quantity of HCAgm derivatives already present in alcohol-free beer (Heineken 0.0 pilsener) and regular beer (Heineken pilsener) was determined, based on the guideline from van Zadelhoff et al. (2024).^25^ The identification of HCAgm derivatives in beer is described in more detail in the Supporting Information.

HCAgm dimers were more abundant in beer compared to the monomers, as the total MS peak area corresponding to HCAgm dimers was approximately seven times higher than that corresponding to HCAgm monomers (Figure S1). From previous work it is known that the MS response of dimers is 1.5 times lower compared to monomers,^25^ indicating that the dimer content is about ten times higher compared to the monomer content. Using the corresponding purified dimers that were obtained previously,^42^ CouAgm-4-*O*-7′/3-8′-DCouAgm (**4**) and FerAgm-4-*O*-7′/3-8′-DFerAgm (**5**) were quantified in the alcohol-free and regular beer (Supporting Information Table S1 and Figure S1). The CouAgm-4-*O*-7′/3-8′-DCouAgm content in alcohol-free and regular beer was 16.2 (±0.5) μg/mL and 15.6 (±0.1) μg/mL, respectively. For FerAgm-4-*O*-7′/3-8′-DFerAgm this was 5.1 (±0.1) μg/mL for alcohol-free beer and 4.6 (±0.0) μg/mL for regular beer.

#### 3.3.3 HCAgm Dimers Obtained via Biomimetic Oxidative Coupling Inhibit Wild Yeast in Beer

In addition to the barley rootlet dimer pools, several individual HCAgm dimers (**4**–**10**, Figure 1) that were obtained via biomimetic HRP/H_2_O_2_ oxidative coupling of CouAgm (**1**) and FerAgm (**2**) as described previously^42^ were included in the next set of experiments. This set of HCAgm dimers was selected because it included CouAgm-4-*O*-7′/3-8′-DCouAgm (**4**) and FerAgm-4-*O*-7′/3-8′-DFerAgm (**5**), derivatives of which were abundant in the pools (Figure 2), as well as several HCAgm dimers with other linkage types that were previously shown to be present in barley (*H. vulgare*) and other *Hordeum* species.^22^ The ferulic acid dimers (**11**–**15**, Figure 1) were not included in further experiments because **11**–**15** were not found to be naturally present in the pools, **12**–**15** did not show any antiyeast activity in the previous experiment (Table 2), and **11** has never been reported as a natural product in *Hordeum* species or other plants.

When testing in standardized media at 5 log_10_ CFU/mL, the only compound for which a MIC was found was FerAgm-4-*O*-7′/3-8′-DFerAgm (**5**) at 250 μg/mL at both pH 4.4, the pH of beer, and 6.5, the pH of the broth (Table 3). The additional set consisted of 2-7′/8-8′-linked and 4-*O*-8′-linked FerAgm (**6** and **7**); and 2-7′/8-8′-linked, 8-8’/9-*N*-7′-linked, and 8-8′-linked SinAgm (**8**, **9**, and **10**).^42^ No inhibition was found for any of these compounds. It is surprising that the antiyeast activity is limited to FerAgm-4-*O*-7′/3-8′-DFerAgm (**5**), as the other tested compounds share the majority of their structural features, such as the charged agmatine moiety, substitution with methoxy groups, and/or the same linkage type. The potency of FerAgm-4-*O*-7′/3-8′-DFerAgm (**5**) is not pH-dependent. Based on the analysis by LC–MS, compound **5** obtained via both synthetic approaches was identical. However, the antimicrobial activity of **5** differed in these experiments (Table 3) compared to the previous experiments (Table 2), which was possibly due to differences in the synthesis or purification. Furthermore, when combining the results of both sets of experiments (Tables 2 and 3), it becomes clear that enhancement of antiyeast activity is dependent on the linkage type formed upon oxidative coupling.

**Table 3:** Overview of the Hydroxycinnamoylagmatine (HCAgm) Monomers, HCAgm Biomimetic Oxidative Coupling Products, and Barley Rootlet Dimer Pools Tested for Their Anti-*S. cerevisiae* subsp. *diastaticus* Activity in Standardised Medium (YPD Broth) at pH 4.4 and 6.5, and in Beer Media (i.e. Alcohol-Free and Regular Beer Broths)^b^

	MIC (μg/mL)			
	standardised media		beer media	
	YPD broth pH 4.4 5 log_10_ CFU/mL	YPD broth pH 6.5 5 log_10_ CFU/mL	alcohol-free beer pH 4.3 1 log_10_ CFU/mL	regular beer pH 4.3 1 log_10_ CFU/mL
HCAgm monomers				
coumaroylagmatine (**1**)	>250	>250	>500	N.t
feruloylagmatine (**2**)	>250	>250	>500	N.t
sinapoylagmatine (**3**)	>250	>250	>500	N.t
HCAgm dimers from biomimetic oxidative coupling				
CouAgm-4-*O*-7′/3-8′-DCouAgm (**4**)	>250	>250	46 (30)^a^	75 (59)^a^
FerAgm-4-*O*-7′/3-8′-DFerAgm (**5**)	250	250	130 (125)^a^	255 (250)^a^
mixture **4**: **5** (4:1 ratio)	N.t	N.t	51 (30)^a^	80 (60)^a^
FerAgm-2-7′/8-8′-DFerAgm (**6**)	>250	N.t	N.t	N.t
FerAgm-4-*O*-8′-DFerAgm (**7**)	>250	N.t	N.t	N.t
SinAgm-2-7′/8-8′-DSinAgm (**8**)	>250	N.t	N.t	N.t
SinAgm-8-8′/9-*N*-7′-DSinAgm (**9**)	>250	N.t	N.t	N.t
SinAgm-8-8′-SinAgm (**10**)	>250	N.t	N.t	N.t
barley rootlet dimer pools				
glycosylated-hydroxylated (**A**)	>250	N.t	>250	N.t
glycosylated (**B**)	>250	>250	>250	>250
hydroxylated (**C**)	N.t	N.t	N.t	N.t
nonsubstituted (**D**)	>250	>250	>1000	>250

a No viable cells were detected at this concentration. N.t.: not tested.

b In beer, the total concentration of dimers present in the beer broth plus added dimer(s) is given, with the added concentration of dimer(s) specified in parentheses.

To translate the results obtained in standardized media to the application in beer, yeast inhibition was studied in broths produced from alcohol-free and regular beer. As alcohol-free and regular beer contained HCAgms, the concentration of the two dimers present in the beer (see Section 3.3.2) was taken into account in the calculation of the final concentrations of the compounds in the MIC values reported in Table 3. An important difference with the antiyeast experiment in standardized media is the initial inoculum size used (i.e., 1 log_10_ CFU/mL). The MIC values in the beer, as well as those from the experiments in standardized media, are provided in Table 3.

The inhibition of wild yeast in alcohol-free beer was compared to regular beer in order to determine the effect of the presence of alcohol on the MIC. The CFU/mL after 18 h incubation in alcohol-free beer broth was 10^2^ times higher compared to yeast grown in regular beer. This indicated lower vitality of the yeast in the regular beer, which is in line with reports on inhibited cell growth and cell division in the presence of alcohol.^51^ Even though yeast vitality in regular beer was lower, the MIC obtained was higher compared to the alcohol-free beer. This could possibly be due to the yeasts stress response to alcohol, resulting in altered membrane composition and fluidity.^52^ The altered membrane may inhibit these compounds’ diffusion through or interaction with the membrane, which may be important for their antiyeast mode of action, thus possibly explaining the higher MIC in regular beer. Alternatively, compositional differences between the two beers, although they are expected to be minimal, could affect the yeast’s susceptibility to the compounds.

Noteworthily, CouAgm-4-*O*-7′/3-8′-DCouAgm (**4**) had a lower MIC than FerAgm-4-*O*-7′/3-8′-DFerAgm (**5**) in beer, whereas in YPD broth only **5** was found to inhibit yeast growth. It was also surprising that the dimers obtained via biomimetic oxidative coupling inhibited yeast growth, whereas no inhibition was observed for the nonsubstituted barley rootlet dimer pool (**D**). The nonsubstituted barley rootlet dimer pool (**D**) is a mixture of the two tested synthesized dimers, CouAgm-4-*O*-7′/3-8′-DCouAgm (**4**) and FerAgm-4-*O*-7′/3-8′-DFerAgm (**5**), and the heterodimer of CouAgm and FerAgm. At the tested concentration of 250 μg/mL of pool **D**, the concentration of **4** in this pool is 2.8 times higher than the MIC found for pure **4** obtained via HRP/H_2_O_2_ oxidative coupling of CouAgm. Thus, it is surprising that dimer pool **D** was not active at all. To verify this finding, a 4-fold higher concentration (1 mg/mL) of the pool **D** was also tested, but even at this concentration no inhibition was observed (Figure 3).

**Figure 3:**
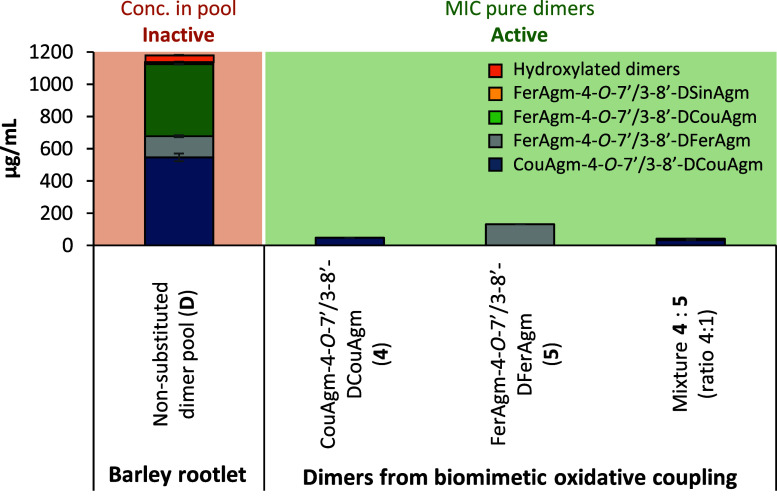
Concentration of HCAgm dimers in 1 mg/mL of barley rootlet dimer pool (orange shading), which was inactive against wild yeast. MIC values of pure HCAgm dimers from biomimetic HRP/H_2_O_2_ oxidative coupling, which were active against wild yeast (green shading) when tested in single compound systems or in a 4:1 mixture of the two dimers.

As is evident from Figure 3, the concentration of **4** or **5** in 1 mg/mL of pool **D** far exceeds the concentrations at which activity was observed for pure **4** and **5** that were obtained via HRP/H_2_O_2_ oxidative coupling of the HCAgm monomers. The concentration of the dimers in this pool was based on semiquantification based on MS peak area and a calibration curve of CouAgm-4-*O*-7′/3-8′-DCouAgm (**4**). Thus, the amount of dimers present in the dimer pool could be overestimated, as the total amount of dimer quantified adds up to 118% of the extract. Nonetheless, the results indicate that even at an approximately ten times higher concentration of CouAgm-4-*O*-7′/3-8′-DCouAgm (**4**) in the barley rootlet dimer pool compared to pure **4**, no inhibition of yeast was observed. To investigate whether any antagonistic effects between **4** and **5** may play a role in this observation, the composition of pool **D** was mimicked by combining **4** and **5** in a 4:1 ratio, which is similar the approximate ratio in which they are present in the pool. This mixture was tested at the same concentration as pool **D**, as well as at lower concentrations. The dimer mixture had a MIC similar to the MIC of **4** on its own, indicating that combining the two dimers does not lead to an antagonistic effect that could explain the lack of inhibition by pool **D**. The nonsubstituted barley rootlet dimer pool (**D**) consisted of at least 50% (*w*/*w*) CouAgm-4-*O*-7′/3-8′-DCouAgm (**4**) and FerAgm-4-*O*-7′/3-8′-DFerAgm (**5**). The remaining part of this pool mostly consisted of FerAgm-4-*O*-7′/3-8′-DCouAgm. The presence of the heterodimer could interfere with the antimicrobial activity, but this is expected to be unlikely.

Overall, these results suggest that dimers produced via biomimetic HRP/H_2_O_2_ oxidative coupling are active, whereas the dimers produced in planta are not. A possible explanation is that the activity is dependent on the stereochemistry of the dimers. In fact, dimers produced via HRP/H_2_O_2_ oxidative coupling are obtained as racemates, whereas the naturally occurring dimers are optically active compounds characterized by *C*-7′*S* and *C*-8′*S* absolute configurations.^29^ The obtained results could be explained considering different potencies for the two enantiomers, as was also previously observed for enantiomers of other natural products,^53,54^ or even more interestingly a possible enantioselective interaction with a still unknown biological target. As it would not be expected that barley selectively produces the inactive enantiomer, the latter option seems more plausible. It is likely that this wild yeast is not the intended target of natural HCAgm dimers.

### 3.4 Exploring the Broader Antimicrobial Activity of Natural Phenolamides

To explore whether the intended natural target(s) of natural HCAgm dimers may be microorganisms other than wild yeast, the antibacterial and antimold activity of the barley rootlet dimer pools, the three HCAgms (i.e., CouAgm, FerAgm, and SinAgm), and compounds **4**–**10**, were tested. Considering the aim of the current work, the Gram-positive bacterium *L. brevis*, a common beer spoilage bacterium, was selected for these exploratory experiments.^8,55^ For assessment of the antimold activity and spore formation inhibiting activity, the molds *A. niger* and *Penicilium commune* were selected, because species from the genera *Aspergillus* and *Penicillium* were reported to be beer spoilage organisms.^7,8^

None of the tested pools or compounds inhibited growth of *L. brevis* (initial inoculum 5 log_10_ CFU/mL) up to a concentration of 250 μg/mL. These results may indicate that HCAgms and their dimers are not effective as antibacterial agents, although screening their potency against a wider array of bacteria would be necessary to verify this result. Moreover, it should be noted that, similar to the yeast strain used, this bacterial strain was a brewery isolate that could already have gained resistance to HCAgm derivatives. No inhibition of mold growth or spore formation was observed at concentrations up to 10 mg/mL. Thus, the previously reported antimold activity of HCAgm dimers, including CouAgm-4-*O*-7′/3-8′-DCouAgm (Table 1), against *A. niger* and *P. commune* could not be reproduced, despite all tested molds belonging to the same phylum (i.e., Ascomycota) and subdivision (i.e., Pezizomycotina). For future studies, it would be recommended to verify these results by testing against at least one of the strains for which inhibition was previously reported (Table 1).

In conclusion, HCAgms and dimers thereof were found not to possess any activity against the common beer spoilage bacterium *L. brevis* or the molds *A. niger* and *P. commune*.

### 3.5 Future Perspectives of Phenolamides as Natural Antimicrobials

A diverse set of ferulic acid and feruloylagmatine (FerAgm) dimers (**5** and **12**–**15**) was obtained via HRP/H_2_O_2_ oxidative coupling of ethylferulate followed by amidation with protected agmatine. This strategy provided ferulic acid dimers, including Fer-3-8′-decarboxyFer (poacic acid; **15**) and HCAgm dimers, including FerAgm-4-*O*-7′/3-8′-DFerAgm (*rac*-hordatine C; **5**). Only **5** and the newly synthesized derivative with a stilbenoid backbone (**11**), which was formed via amidation of poacic acid with agmatine, inhibited the wild yeast *S. cerevisiae* subsp. *diastaticus*. Thus, functionalizing hydroxycinnamic acid dimers with agmatine can be used as a tool to enhance their antimicrobial potency, which warrants further exploration of the potential to more broadly improve the functionality of these compounds by amidation with agmatine and other natural (poly)­amines, such as putrescine or tyramine. Additionally, the presented results show that oxidative coupling of HCAgms also increases their antiyeast activity.

Additionally, four barley rootlet dimer pools and a set of HCAgm dimers (**4**–**10**) that were obtained via a previously developed biomimetic strategy in which dimers of HCAgm dimers were formed via HRP/H_2_O_2_ oxidative coupling of HCAgm monomers that were produced via amidation of nonprotected hydroxycinnamic acids were studied.^20,42,43^ In standardized media (pH 4.4 and 6.5, initial inoculum 5 log_10_ CFU), inhibition of *S. cerevisiae* subsp. *diastaticus* was only observed for one compound, namely FerAgm-4-*O*-7′/3-8′-DFerAgm (**5**) at a concentration of 250 μg/mL. None of the other compounds or pools were able to inhibit this wild yeast in standardized media. However, when tested in beer with an initial inoculum size that mimics realistic contamination of beer (1 Log_10_ CFU/mL), both CouAgm-4-*O*-7′/3-8′-DCouAgm (**4**) and FerAgm-4-*O*-7′/3-8′-DFerAgm (**5**) inhibited growth of the yeast. To explore whether microorganisms other than wild yeast may be the intended target(s) of natural HCAgms, the activity of the pools and compounds **4**–**10** was tested against the beer spoilage bacterium *L. brevis* and the molds *Penicillium commune* and *A. niger*. However, no antibacterial or antimold activity was found for any of the compounds or pools tested.

To conclude, two HCAgm dimers formed via biomimetic oxidative coupling were active against *S. cerevisiae* subsp. *diastaticus*, a wild yeast that is involved in spoilage of (alcohol-free) beer, which makes further exploration of these compounds for application as preservatives in beer interesting. Although no definite conclusions on the underlying mechanisms could be drawn, a possible explanation for the observed difference in activity between the biomimetically produced dimers and the dimers naturally present in the barley rootlet dimer pools is related to difference in their stereochemistry, and thus their interaction with their biological target in the yeast. The presented findings call for follow-up studies on the effect of the stereochemistry of HCAgm dimers on their antimicrobial activity, as well as testing their activity against a wider diversity of microorganisms. Furthermore, it would be prudent to further investigate the natural function of HCAgms and their dimers from barley, including exploration of novel potential biological targets of these compounds.

Although the applicability of HCAgm dimers obtained from barley rootlets is expected to be limited for preservation of alcohol-free beer, there may be opportunities to use these natural compounds or their biomimetically produced analogues in other food products or in applications outside food. Prior to application of the biomimetically produced 4-*O*-7′/3-8′-linked dimers as antiyeast preservatives, some challenges should be considered. First, the food safety and sensory attributes of the compounds should be studied. Regarding safety, since HCAgm dimers are already present in barley and beer, and are thus regularly consumed, these dimers could be generally regarded as safe (GRAS).^56^ It is, however, of importance to test all the enantiomers for their toxicity, since it is known that bioactivity and toxicity can greatly vary between enantiomers.^57^

## Supplementary Material



## Notes

Characterization of HCAgm derivatives in beer; RP-UHPLC-PDA-IT-MS base peak chromatograms (*m*/*z* 200–1500) in the positive ionization mode for alcohol-free beer and regular beer, and the monomeric and dimeric composition of alcohol-free and regular beer based on MS peak area; chromatographic and spectral data with (tentative) identification of the 20 most abundant HCAgm derivatives in alcohol-free and regular beer; NMR data for synthesized compounds (PDF)
