# Supplementary material for: Natural Strategies to Preserve Alcohol-Free Beer: Phenolamide Dimers with Anti-Yeast Potential
Source: J Agric Food Chem. 2026 Feb 22;74(8):6754–66. doi: 10.1021/acs.jafc.5c09118 (PMC12964544; doi:10.1021/acs.jafc.5c09118)
Supplement: Supplementary file 1 [file jf5c09118_si_001.pdf]

Supporting information

Natural Strategies to Preserve Alcohol-Free Beer: Phenolamide Dimers with Anti-Yeast Potential

Annemiek van Zadelhoff[1], Denise Dozio[2], Francesca Annunziata[2], Aziza Caccia[2], Sabrina Dallavalle[2], Yingyu Zhou[1], Sarah van Dinteren[1], Jean-Paul Vincken[1], Andrea Pinto[2,*], Wouter J.C. de Bruijn[1,*]

[1] Laboratory of Food Chemistry, Wageningen University, Bornse Weilanden 9, 6708 WG Wageningen, The Netherlands
[2] Department of Food, Environmental and Nutritional Sciences (DeFENS), University of Milan, 20133 Milan, Italy

* Corresponding authors:
Wouter J.C. de Bruijn (wouter.debruijn@wur.nl)
Andrea Pinto (andrea.pinto@unimi.it)

Table of contents

**Characterization of HCAgm derivatives in beer**	Page 2
**Figure S1**	Page 3
**Table S1**	Page 4
**NMR data for synthesized compounds**	Page 5-7

Characterization of HCAgm derivatives in beer

The chromatographic profiles of the alcohol-free and the regular beer were almost identical (**Figure S1**). The twenty most abundant HCAgm derivatives were (tentatively) identified using the previously designed identification guideline[26] and are presented in **Table S1**. No new HCAgm derivatives were detected in beer and the compounds detected were in line with previous reports on HCAgms and dimers thereof in beer.[25, 58]

HCAgm dimers were more abundant in beer compared to the monomers, with an approximately seven times higher total MS peak area for the dimers. Both non-substituted and hydroxylated HCAgms were the main monomers detected in both alcohol-free and regular beer, whereas in beer brewing by-products the majority of the monomers present were hydroxylated on at least one of the agmatine moieties. The dimers in beer were predominantly non-substituted, which is also different from the beer brewing by-products, in which the majority of dimers was detected with at least one hexosyl residue. In our previous work,[26] five new compound types were characterized, of which three were also detected in the alcohol-free and the regular beer, namely type 1, type 4, and type 5 compounds.

A clear difference between the barley rootlet extracts and the beer samples is that for the beer most of the abundant compounds detected are non-glycosylated, whereas the majority of compounds in barley rootlet extracts were glycosylated.[26] The most abundant HCAgm derivatives in beer were also detected in the barley rootlet extracts, except for CouAgm-4-*O*-7′/3-8′-(4′$_{\text{Hex-Hex-Hex}}$)-DCouAgm, a dimer with three hexose units attached. Among the less abundant compounds in beer (data not shown in **Table S1**), the CouAgm homodimer was detected with up to five hexose units. The CouAgm-FerAgm heterodimer was detected in beer with up to four hexose units attached and FerAgm-4-*O*-7′/3-8′-DFerAgm with a maximum of three. The relative concentration of hexosylated compounds is lower but the number of hexose residues in the hexosyl chain was higher in beer compared to barley. These longer hexosyl chains attached to HCAgms in beer compared to barley is in line with a previous report on barley and beer analysis, in which dimers in barley had hexosyl chains with up to three hexosyl residues and those in beer up to nine.[25] It is unknown why the number of hexosyl residues attached to dimers is higher in beer compared to that in barley. In beer, the type 2 compounds, as classified in our previous work,[26] were not detected, even though this type of compounds were among the abundant compounds detected in the malt. Since the glycosylated dimers were the most abundant compounds in malt and not in beer, the difference in the HCAgm derivative composition between barley and beer is probably introduced during the brewing process.

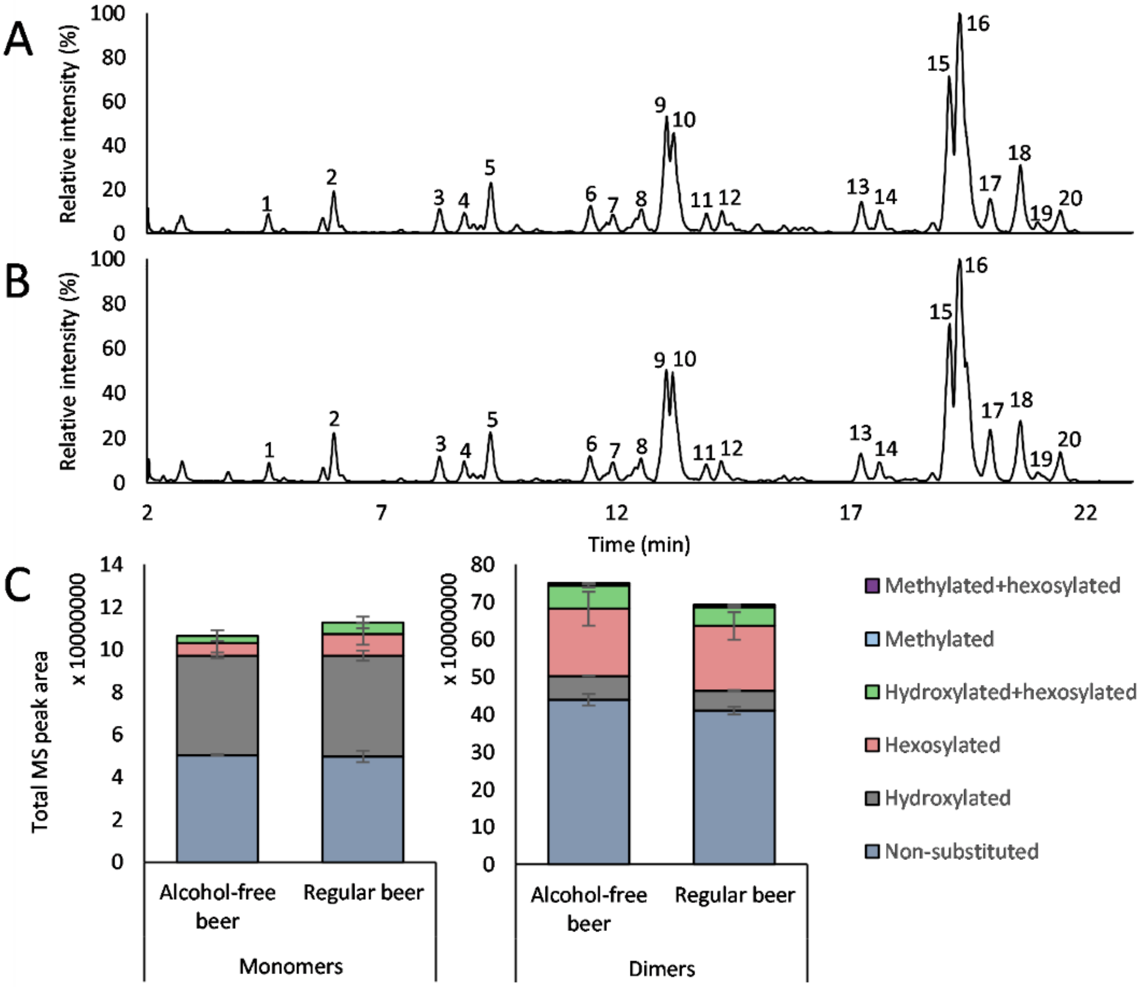

Figure S1. RP-UHPLC-PDA-IT-MS base peak chromatograms (*m/z* 200–1,500) in the positive ionization mode for alcohol-free beer (A) and regular beer (B), and the monomeric and dimeric composition of alcohol-free and regular beer based on MS peak area (C). Methylated refers to substitution of at least one guanidino group with a methyl, hydroxylated refers to presence of at least one hydroxyl group on *C*-12$^{(')}$ (i.e. on the agmatine moiety), and hexosylated refers to substitution of *C*-4$^{(')}$ with at least one hexose.

Supporting information with “Natural Strategies to Preserve Alcohol-Free Beer: Phenolamide Dimers with Anti-Yeast Potential” by van Zadelhoff, et al.

Table S1. Chromatographic and spectral data with (tentative) identification of the twenty most abundant HCAgm derivatives in alcohol-free and regular beer.

Peak nr.	RT (min)	Ionisation	*m/z*	MS^2 (relative intensity)	(Tentative) identification
1	4.60	$[M+H]^+$	439	260 (100), 277 (77), 217 (32), 422 (32), 147 (27), 261 (15), 278 (13), 218 (13)	(4_{Hex})-CouAgm [a]
2	5.98	$[M+H]^+$	293	275 (100), 276 (37), 295 (18), 147 (16)	CouAgh [a]
3	8.24	$[M+H]^+$	323	305 (100), 306 (36), 177 (25), 289 (11)	FerAgh [a]
4	8.77	$[M+2H]^{2+}$	388	307 (100), 379 (21), 298 (15), 441 (7), 596 (4)	FerAgh-4-*O*-7′/3-8′-($4'_{Hex}$)-DCouAgh [a]
5	9.33	$[M+H]^+$	277	260 (100), 114 (16), 217 (15), 261 (13), 115 (12)	CouAgm
6	11.46	$[M+2H]^{2+}$	380	299 (100), 290 (8), 371 (8), 425 (6), 580 (5)	($_{OH}$)-FerAgm-4-*O*-7′/3-8′-($4'_{Hex}$)-DCouAgm [a]
7	11.94	$[M+H]^+$	307	298 (100), 441 (44), 268 (38), 321 (26), 147 (16), 442 (13), 173 (13), 484 (11)	FerAgm
8	12.55	$[M+2H]^{2+}$	519	276 (100), 438 (77), 357 (48), 713 (29), 551 (21), 395 (11)	CouAgm-4-*O*-7′/3-8′-($4'_{Hex-Hex-Hex}$)-DCouAgm [a]
9	13.09	$[M+2H]^{2+}$	372	291 (100), 292 (7), 363 (7), 281 (6), 425 (6), 564 (4)	FerAgm-4-*O*-7′/3-8′-($4'_{Hex}$)-DCouAgm [a]
10	13.23	$[M+2H]^{2+}$	357	276 (100), 367 (7), 277 (6), 348 (5)	CouAgm-4-*O*-7′/3-8′-($4'_{Hex}$)-DCouAgm [a]
11	13.93	$[M+2H]^{2+}$	307	298 (100), 441 (44), 268 (38), 321 (26), 147 (16), 442 (13), 173 (13), 484 (11)	FerAgh-4-*O*-7′/3-8′-DCouAgh [a]
12	14.26	$[M+2H]^{2+}$	468	306 (100), 387 (10)	FerAgm-4-*O*-7′/3-8′-($4'_{Hex-Hex}$)-DFerAgm [a]
13	17.23	$[M+2H]^{2+}$	299	290 (100), 441 (96), 260 (71), 321 (34), 157 (25), 442 (25), 131 (20), 269 (19), 468 (14), 423 (14), 281 (10)	($_{OH}$)-FerAgm-4-*O*-7′/3-8′-DCouAgm [a]
14	17.62	$[M+2H]^{2+}$	284	275 (100), 245 (68), 411 (66), 254 (25), 291 (25), 131 (17), 412 (16), 211 (16), 267 (14), 393 (14), 438 (13), 263 912), 157 (11), 98 (10)	($_{OH}$)-CouAgm-4-*O*-7′/3-8′-DCouAgm [a]
15	19.10	$[M+2H]^{2+}$	291	282 (100), 425 (95), 261 (80), 157 (31), 426 (25), 451 (18), 270 (13), 321 (12), 131 (11)	FerAgm-4-*O*-7′/3-8′-DCouAgm [a]
16	19.32	$[M+2H]^{2+}$	276	246 (100), 267 (99), 395 (55), 396 (15), 255 (13), 157 (11), 421 (11)	CouAgm-4-*O*-7′/3-8′-DCouAgm
17	19.97	$[M+2H]^{2+}$	291	282 (100), 425 (96), 261 (84), 157 (29), 426 (28), 451 (18), 270 (13), 321 (11)	FerAgm-4-*O*-7′/3-8′-DCouAgm [a]
18	20.62	$[M+2H]^{2+}$	306	297 (100), 455 (94), 276 (86), 157 (32), 481 (26), 456 (25), 351 (12), 131 (12), 285 (11)	FerAgm-4-*O*-7′/3-8′-DFerAgm
19	20.99	$[M+2H]^{2+}$	291	282 (100), 261 (96), 425 (57), 426 (18), 451 (15), 157 (13), 270 (130), 321 (11)	CouAgm-4-*O*-7′/3-8′-DFerAgm [a]
20	21.46	$[M+2H]^{2+}$	306	297 (100), 455 (98), 276 (87), 157 (32), 456 (28), 481 (27), 131 (13), 351 (12), 285 (11)	FerAgm-4-*O*-7′/3-8′-DFerAgm

[a]Tentatively identified based on MS fragmentation patterns.

NMR data for synthesized compounds

Compound **5**

1H NMR (400 MHz, methanol-d_4) δ 8.57 – 8.45 (m, 1H), 7.49 (d, *J* = 15.5 Hz, 1H), 7.19 (s, 1H), 7.03 – 6.93 (m, 2H), 6.82 (s, 2H), 6.49 (d, *J* = 15.5 Hz, 1H), 5.97 (d, *J* = 8.1 Hz, 1H), 4.25 (d, *J* = 8.1 Hz, 1H), 3.94 (s, 3H), 3.85 (s, 3H), 3.43 – 3.33 (m, 4H), 3.28 – 3.17 (m, 4H), 1.72 – 1.58 (m, 8H).

^{13}C NMR (101 MHz, methanol-d_4) δ 171.83, 167.75, 157.26, 157.21, 149.96, 147.93, 146.86, 144.82, 140.35, 131.15, 129.07, 128.08, 118.64, 118.08, 116.88, 114.96, 111.46, 109.16, 88.57, 57.32, 55.42, 55.04, 40.69, 40.61, 38.62, 38.44, 26.72, 26.37, 25.80.

Compound **11**

^{1}H NMR (400 MHz, methanol-d_4) δ 7.51 (d, *J* = 15.7 Hz, 1H), 7.39 (d, *J* = 1.7 Hz, 1H), 7.30 (d, *J* = 16.5 Hz, 1H), 7.15 (m, 2H), 7.05 (d, *J* = 1.7 Hz, 1H), 7.00 (dd, *J* = 8.2, 1.8 Hz, 1H), 6.80 (d, *J* = 8.1 Hz, 1H), 6.52 (d, *J* = 15.7 Hz, 1H), 3.94 (s, 3H), 3.93 (s, 3H), 3.41 – 3.34 (m, 2H), 3.25 (m, 2H), 1.66 (m, 4H).

^{13}C NMR (101 MHz, methanol-d_4) δ 168.01, 157.26, 148.05, 147.83, 146.29, 145.84, 141.10, 130.11, 129.20, 126.06, 124.73, 119.92, 119.77, 119.12, 117.38, 114.95, 108.94, 107.54, 55.17, 54.99, 40.69, 38.33, 26.44, 25.75.

Compound **12**

^{1}H NMR (400 MHz, acetone-d_6) δ 7.64 (d, J = 2.0 Hz, 1H), 7.40 (d, J = 1.9 Hz, 1H), 7.24 (dd, J = 8.3, 2.0 Hz, 1H), 7.00 – 7.06 (bs, 1H), 6.92 (d, J = 8.2 Hz, 1H), 6.88 – 6.82 (m, 2H), 5.78 (d, J = 2.8 Hz, 1H), 4.35 (t, J = 2.4 Hz, 1H), 3.90 (s, 3H), 3.85 (t, J = 7.1 Hz, 3H).

^{13}C NMR (101 MHz, acetone-d_6) δ 171.10, 170.71, 149.30, 147.75, 147.64, 147.09, 139.67, 131.47, 125.70, 125.67, 119.51, 118.33, 115.31, 115.15, 113.09, 109.30, 80.32, 55.40, 53.05.

Compound **13**

^{1}H NMR (400 MHz, acetone-d_6) δ 7.66 (d, *J* = 15.9 Hz, 1H), 7.36 (s, 1H), 7.34 (s, 1H), 7.12 (d, *J* = 1.9 Hz, 1H), 6.95 (dd, *J* = 8.2, 1.9 Hz, 1H), 6.86 (d, *J* = 8.2 Hz, 1H), 6.43 (d, *J* = 15.9 Hz, 1H), 6.07 (d, *J* = 7.8 Hz, 1H), 4.46 (d, *J* = 7.8 Hz, 1H), 3.95 (s, 3H), 3.86 (s, 3H).

^{13}C NMR (100 MHz, acetone-d_6) δ 171.37, 167.45, 147.94, 147.73, 146.72, 146.07, 145.16, 130.05, 129.83, 126.13, 124.59, 120.29, 119.99, 119.74, 115.66, 115.11, 109.27, 108.28, 55.69, 55.39.

Compound **14**

^{1}H NMR (400 MHz, acetone-d_6) δ 7.82 (s, 1H), 7.61 (d, J = 15.9 Hz, 1H), 7.40 (d, J = 1.9 Hz, 1H), 7.05 (d, J = 1.9, 1H), 6.88 (dd, J = 8.3, 1.9 Hz, 1H), 6.76 (d, J = 1.9, 1H), 7.64 (d, J = 8.2 Hz, 1H), 6.40 (d, J = 15.9 Hz, 1H), 3.99 (s, 3H), 3.48 (t, J = 7.1 Hz, 3H).

^{13}C NMR (101 MHz, acetone-d_6) δ 168.09, 167.38, 148.31, 148.10, 147.20, 147.02, 144.88, 140.81, 126.84, 126.47, 125.70, 125.50, 124.82, 124.38, 115.42, 114.78, 112.42, 109.42, 55.72, 54.66.

Compound **15**

^{1}H NMR (400 MHz, acetone-d_6) δ 7.66 (d, *J* = 15.9 Hz, 1H), 7.57 (d, *J* = 1.8 Hz, 1H), 7.35 (d, *J* = 1.6 Hz, 2H), 7.26 (d, *J* = 1.8 Hz, 1H), 7.25 (d, *J* = 1.8 Hz, 1H), 7.08 (dd, *J* = 8.2, 1.9 Hz, 1H), 6.86 (d, *J* = 8.2 Hz, 1H), 6.47 (d, *J* = 15.9 Hz, 1H), 3.97 (s, 3H), 3.93 (s, 3H).

^{13}C NMR (101 MHz, acetone-d_6) δ 167.51, 147.94, 147.73, 146.72, 146.07, 145.16, 130.05, 129.83, 126.13, 124.59, 120.29, 119.99, 119.74, 115.66, 115.11, 109.27, 108.28, 55.69, 55.39.’

Compound **16**

^{1}H NMR (400 MHz, acetone-d_6) δ 7.60 (d, J = 15.9 Hz, 1H), 7.35 (d, J = 1.9 Hz, 1H), 7.15 (dd, J = 8.2, 1.9 Hz, 1H), 6.89 (d, J = 8.2 Hz, 1H), 6.40 (d, J = 15.9 Hz, 1H), 4.20 (q, J = 7.1 Hz, 2H), 3.94 (s, 3H), 1.29 (t, J = 7.1 Hz, 3H).

Compound **17**

^{1}H NMR (400 MHz, acetone-d_6) δ 7.64 (d, *J* = 15.9 Hz, 1H), 7.35 (s, 1H), 7.30 (s, 1H), 7.11 (d, *J* = 1.9 Hz, 1H), 6.94 (dd, *J* = 8.2, 1.9 Hz, 1H), 6.86 (d, *J* = 8.2 Hz, 1H), 6.44 (d, *J* = 15.9 Hz, 1H), 6.05 (d, *J* = 8.0 Hz, 1H), 4.45 (d, *J* = 8.0 Hz, 1H), 4.29 (q, *J* = 7.0 Hz, 2H), 4.21 (q, *J* = 7.0 Hz, 2H), 3.96 (s, 3H), 3.86 (s, 3H), 1.37 – 1.26 (m, 6H).

^{13}C NMR (101 MHz, acetone-d_6) δ 170.23, 166.41, 150.12, 147.68, 147.07, 144.96, 144.37, 131.22, 128.59, 126.53, 119.33, 118.06, 115.81, 114.92, 112.38, 109.86, 87.48, 61.34, 59.70, 55.61, 55.43, 55.19, 13.62.

Compound **18**

^{1}H NMR (400 MHz, chloroform-d_1) δ 8.45 (s, 1H), 3.38 (t, *J* = 6.7 Hz, 2H), 2.91 (t, *J* = 6.3 Hz, 2H), 1.73 – 1.60 (m, 4H), 1.50 (s, 18H).

Compound **19**

^{1}H NMR (400 MHz, acetone-d_6) δ 11.60 (s, 1H), 8.45 (s, 1H), 8.10 (s, 1H), 7.70 (s, 1H), 7.50 (d, *J* = 15.6 Hz, 1H), 7.45 (d, *J* = 1.7 Hz, 1H), 7.32 (t, *J* = 17.9 Hz, 2H), 7.24 (d, *J* = 1.5 Hz, 1H), 7.09 (d, *J* = 1.7 Hz, 1H), 7.07 (dd, *J* = 8.2, 1.8 Hz, 1H), 6.85 (d, *J* = 8.1 Hz, 1H), 6.58 (d, *J* = 15.6 Hz, 1H), 3.93 (s, 3H), 3.92 (s, 3H), 3.51 – 3.40 (m, 4H), 1.75 – 1.58 (m, 4H), 1.53 (s, 9H), 1.45 (s, 9H).

^{13}C NMR (101 MHz, acetone-d_6) δ 165.43, 165.35, 163.75, 156.00, 152.92, 147.77, 146.67, 145.23, 139.53, 130.03, 129.56, 126.86, 124.50, 120.21, 119.98, 119.67, 118.71, 115.11, 109.24, 108.20, 82.76, 78.00, 55.56, 55.38, 40.14, 40.02, 38.68, 38.55, 27.62, 27.25, 26.70, 26.65.

Compound **20**

^{1}H NMR (400 MHz, acetone-d_6) δ 11.73 – 11.61 (m, 1H), 8.43 – 8.29 (m, 1H), 7.98 (s, 2H), 7.73 – 7.59 (m, 1H), 7.53 – 7.42 (m, 1H), 7.39 – 7.29 (m, 1H), 7.15 – 7.13 (m, 1H), 7.13 – 7.11 (m, 1H), 7.04 – 7.02 (m, 1H), 6.90 – 6.86 (m, 1H), 6.85 (s, 1H), 6.58 – 6.47 (m, 1H), 6.06 – 6.00 (m, 1H), 4.34 – 4.23 (m, 1H), 3.90 (s, 2H), 3.85 (s, 2H), 3.52 – 3.29 (m, 6H), 1.64 (s, 6H), 1.53 (s, 13H), 1.44 (d, *J* = 6.8 Hz, 13H).

^{13}C NMR (101 MHz, acetone-d_6) δ 169.73, 165.30, 163.73, 161.84, 156.05, 152.94, 149.63, 147.62, 146.85, 144.67, 139.23, 131.86, 129.26, 128.70, 119.75, 118.94, 116.37, 114.99, 112.10, 109.68, 90.64, 88.35, 82.77, 77.97, 57.35, 55.53, 40.15, 40.06, 39.03, 38.68, 35.24, 27.61, 27.26.
